# Validation of cleaning process of cardiac catheters and reuse in the hemodynamics sector of a hospital in Ponta Grossa, Parana, Brazil

**DOI:** 10.1186/1753-6561-5-S6-P214

**Published:** 2011-06-29

**Authors:** M Gaspar, CLD Silva, CRB Bastos, JSD Nascimento, VRD Maia

**Affiliations:** 1 Departamento de Enfermagem e Saúde Pública, Ponta Grossa, Brazil; 2 Universidade Estadual de Ponta Grossa – Paranà, Ponta Grossa, Brazil; 3 Santa Casa de Misericórdia de Ponta Grossa, Ponta Grossa, Brazil; 4 Hospital Regional de Ponta Grossa, Ponta Grossa, Brazil

## Introduction / objectives

Reprocessing single use devices is the process to be applied to hospital/medical objects which allows their reuse; in order to reuse them it is necessary to include steps in order to ensure that the catheters are clean, without organic matter, reducing the microbial load, pyrogens and biofilm. To develop and validate a cleaning protocol for objects from a cardiology sector in a hospital in Ponta Grossa, Parana, Brazil.

## Methods

A quantitative and experimental study undertaken in February and March 2011 with a sample of 72 cardiac catheters. A protocol for cleaning tools and training with the nursing staff has been developed, using as a method to detect organic matter in the lumen, the Hemocheck ®, a tool that can detect from 0ug to 100 ug.

## Results

In the beginning, we observed that the catheters were flushing with 1 (1.38%) of the catheters had 100 ug of matter in the lumen, 5 (6.94%) had 10 ug of dust, 3 (4.16% ) of the catheters showed 1 ug, 11 (15.27%) showed 0.1 ugof organic matter and 52 (72.22%) with 0μg of dirt in the lumen; after 45 days conducting a systematic way of cleaning and tests in random days and times, there was not positivity of dirt in the catheter compared to results found previously.

## Conclusion

The Infection Control Service and Materials Centrer of will need to implement the protocol, giving priority to patient safety, as the critical items such as the hemodynamic, by its nature and we need more care with this item.

## Disclosure of interest

None declared.

